# Screening for Resistance in Selected Tomato Varieties against the Root-Knot Nematode *Meloidogyne incognita* in the Philippines Using a Molecular Marker and Biochemical Analysis

**DOI:** 10.3390/plants11101354

**Published:** 2022-05-19

**Authors:** Roden Carlo M. Lizardo, Marita S. Pinili, Maria Genaleen Q. Diaz, Christian Joseph R. Cumagun

**Affiliations:** 1 Institute of Weed Science, Entomology and Plant Pathology, College of Agriculture and Food Science, University of the Philippines Los Baños, College, Los Baños 4031, Laguna, Philippines; rmlizardo1@up.edu.ph; 2 National Crop Protection Center, College of Agriculture and Food Science, University of the Philippines Los Baños, College, Los Baños 4031, Laguna, Philippines; maripinili@gmail.com; 3 Institute of Biological Sciences, College of Arts and Science, University of the Philippines Los Baños, College, Los Baños 4031, Laguna, Philippines; mqdiaz@up.edu.ph; 4 Molecular Phytopathology and Mycotoxin Research, University of Göttingen, Grisebachstrasse, 637077 Göttingen, Germany

**Keywords:** tomato, resistance screening, *Meloidogyne incognita*, Mi23, biochemical response

## Abstract

Tomato is a high-value vegetable crop widely cultivated in the Philippines, and its production is threatened by various stresses including infection by the root-knot nematode *M. incognita*. In this study, we checked for resistance to *M. incognita* in selected tomato germplasm collections and commercially available varieties using a bioassay method, the molecular marker Mi23 and biochemical analysis. Among the eight varieties tested, none showed a resistant reaction against *M. incognita*. Use of the molecular marker *Mi23* yielded 430 bp in all the tomato varieties screened. Phylogenetic analysis using the neighbor-joining method revealed the clustering of consensus sequence of the varieties tested with the susceptible variety *S. lycopersicum* cv. M82-1-8 and a wild relative, *S. pimpinellifolium* isolate LA2184. The biochemical analysis showed varying responses among the varieties when they were inoculated with *M. incognita*. Increased levels of total antioxidant activity were observed in Diamante Max F1, Ilocos Red and Tm 2016 11-1, while total phenolic content was found to be elevated in Athena, Avatar TY F1 and Rosanna. Increased levels of ascorbic acid were observed in Athena and Avatar TY F1 even at 45 dpi. Even though these varieties showed elevated levels of the abovementioned biochemical parameters related to a resistance reaction, all of them showed highly susceptible reactions. Hence, this study showed that these tomato varieties have no resistance against *M. incognita* and that there is a need to identify other sources of resistance against *M. incongita* and produce resistant tomato cultivars adapted to local conditions.

## 1. Introduction

Tomato (*Solanum lycopersicum* L.) is a vine grown for its edible berry that is consumed raw or cooked. It is one of the most important vegetables cultivated worldwide, with production of 182 million tons grown on a total of 4.8 million hectares. In the Philippines, around 16,491 hectares have been planted to tomato, with production of 218,793 tons [1]. According to the Philippines Statistics Authority (PSA) [2], from 2007 to 2017, tomato production growth averaged 1.23% per year. The major producing areas are Ilocos Norte and Ilocos Sur in Luzon, Iloilo and Cebu in Visayas, and Misamis Oriental and Bukidnon in Mindanao. 

Global and local production of tomato is threatened by various factors, which include the existing local weather, climate change, and the presence of pests and diseases. The presence of root-knot disease in tomato and other vegetable crops has been established in the Philippines. Yield losses due to root-knot nematodes (RKNs) can reach over 30% for susceptible crops, which include tomato, eggplant and melon [3].

Root-knot is caused by plant-parasitic nematodes belonging to the genus *Meloidogyne*, and symptoms include the presence of galls in roots due to parasitism by these sedentary endoparasites, which affects the absorption and transport of water and nutrients from the soil. This leads to stunting, wilting and general chlorosis [4]. In the Philippines, yield losses of 20% to 85% caused by RKNs have been recorded, depending on the location where tomatoes are grown [5]. Among the reported species of *Meloidogyne*, *M. incognita* is the most important in terms of the reported distribution and damage inflicted on the production of various economically important crops, including tomato, in the Philippines [6,7]. 

Various control methods and management strategies can be implemented to avoid economic damage due to RKN infestation. They can include cultural, physical, biological, cropping-based, chemical and resistance methods. However, the use of each method may depend on their suitability to the cropping system and the scale of operation of the farmers. Moreover, some of the methods may not be compatible with each other when implemented together to manage RKN populations in the field. Hence, for controlling soil-borne pathogens such as RKNs, the use of resistant varieties is most practical because of the reduced cost of production, and because it is environmentally sound and compatible with other control methods [3].

Many of the cultivated tomato nowadays carry the *Mi* gene that confers field resistance against *Meloidogyne incognita*, *M. javanica* and *M. hapla* [8,9]. It is a single, dominant gene introgressed into cultivated tomato from the wild relative *Lycopersicon peruvianum* (syn. *S. peruvianum*), which has resistance to RKN [10]. Since then, many varieties have been bred using the F_1_ of these two species and were used as parental lines for nematode resistance breeding programs [11]). Biochemical studies have also been carried out to understand the nature of the resistance response against nematodes, which includes the involvement of plant hormones, antioxidants, phenols, enzymes, pathogen-related proteins and ascorbic acid, among others [12,13,14,15,16].

Screening for resistance in tomato against *M. incognita* has been achieved by using the presence of *the Mi-1* gene as the basis of resistance. A combination of traditional screening methods and molecular markers has been used in order to identify possible lines to be used for breeding and for rapid screening of germplasm for nematode resistance. The SCAR marker Mi23 was recommended to be used for resistance screening against RKN, as it is more reliable and practical compared with other molecular markers such as REX-1 and PMi12 [17,18]. This study made use of the primer pairs found by Seah et al. [19], which are very good at discriminating between resistant (380 bp amplicons) and susceptible (430 bp amplicons) genotypes, and targets chromosome 6 of the tomato genome. In the Philippines, identifying tomato lines to be used for nematode resistance breeding remains a challenge. Nine tomato lines were identified as resistant to both *M. incognita* and *M. javanica* [20]. However, no further studies on resistance among local tomato varieties have been carried out in the Philippines.

This study aimed to identify resistance to *M. incognita* in local germplasm collections and tomato cultivars by using a bioassay method, by detecting the presence of *Mi-1* gene using a molecular marker Mi23, and by assessing the biochemical response of these tomato varieties against RKN infection in relation to their disease reaction.

## 2. Materials and Methods

### 2.1. Preparation of Nematode Cultures

The *M. incognita* isolate was collected from the Institute of Plant Breeding (IPB), College of Agriculture and Food Science (CAFS), University of the Philippines Los Baños (UPLB), Laguna, Philippines. Twenty plastic pots 15 cm in diameter containing a 1 kg mixture of sterile soil, river sand and coir dust (1:1:0.5) were planted with two 2-week-old plants of the susceptible tomato variety Rosanna. The plants were inoculated with galled tomato roots two weeks after transplanting. Adult female nematodes were collected randomly from inoculated tomato roots at 28 days post-inoculation (dpi). The species was identified as *M. incognita* by examining their perineal pattern [21,22]. 

### 2.2. Nematode Extraction and Inoculum Standardization

*M. incognita*-infected tomato plants were uprooted and gently washed with flowing water to remove excess soil. The roots were air-dried at room temperature and the egg masses were collected from the roots manually using fine-tipped tweezers. The collected egg masses were placed in small plastic petri dishes containing tap water and incubated for 24–36 h for hatching. Hatched juveniles were collected, and the required numbers of juveniles were counted on a fabricated acrylic counting dish under a stereomicroscope.

### 2.3. Preparation of Planting Materials for Resistance Screening

Screening of the tomato varieties was conducted in the net house of Plant Nematology, IPB, CAFS, UPLB. In total, eight varieties (Diamante Max F1, Ilocos Red, Rosanna, Avatar TY F1, Athena, Rica, Tm 2016 11-1 Cherry and 2018-54 Cherry Tm LTB) of tomato were screened for resistance against the root-knot nematode *M. incognita*. These varieties were selected on the basis of their commercial importance and records of having resistance against other diseases. The tomato variety Rosanna, previously utilized for mass production of the nematodes, was used as the susceptible control. The seeds of Ilocos Red, Rosanna, Rica, Tm 2016 11-1 Cherry and 2018-54 Cherry LTB were sourced from the National Plant Genetic Resource Laboratory (NPGRL), IPB, CAFS, UPLB, while seeds of Diamante Max F1, Avatar TY F1 and Athena were obtained from a nearby agricultural store. Seeds were sown in trays containing sterile soil and were transplanted to plastic pots 15 cm in diameter filled with soil as previously described for nematode culture. There were 12 replications for each variety in a completely randomized design (CRD). Two weeks after transplanting, 6 tomato plants per variety were inoculated with freshly hatched J2 of *M. incognita* at a rate of 1000 J2/pot, as specified by [23], into three holes near the root zone and were covered with the same soil mixture. Plants were watered regularly to keep soil moist, and an appropriate rate of fertilizer was applied throughout the duration of the experiment.

### 2.4. Resistance Screening Using a Bioassay Method 

Plants were uprooted 45 days post-inoculation (dpi) and were washed with flowing tap water to remove the soil. The roots were air-dried at room temperature. Plant parameters such as plant height, plant fresh weight, shoot weight, root length and root weight were collected. The percentage of reduction was computed using the plant parameters measured from the inoculated and uninoculated pots. The average number of root galls per variety was determined and compared with a gall index (GI). Gall indices were assessed through a visual rating based on the six-point rating scale (0–5) devised by Taylor and Sasser [24] (0 = no gall or no infection (immune; I); 1 = 1–2 galls (highly resistant; HR); 2 = 3–10 galls (resistant; R); 3 = 11–30 galls (moderately resistant; MR); 4 = 31–100 galls (susceptible; S), and 5 = 100 or more galls (highly susceptible; HS)]. 

### 2.5. Statistical Analysis

The percentage of reduction in plant height, plant fresh weight, shoot weight, root weight, number of galls and the assessed galling index were analyzed by one-way ANOVA with post-hoc Tukey’s honestly significant difference test (*p* ≤ 0.05) using R Commander (Rcmdr) [25] in RStudio. On the other hand, since the data from the assessed gall indices were non-parametric, the Kruskal–Wallis H-test was performed, followed by Dunn’s test.

### 2.6. Marker Analysis Using Mi23

DNA samples were extracted from tomato leaves of each variety using the cetryltrimethylammonium bromide (CTAB) extraction method as described by Doyle and Doyle [26] with modifications. Molecular screening for resistance against *M. incognita* was caried out using the marker Mi23 [19]. DNA amplification was performed using a G-Storm GS04822 Thermal Cycler (Gene Technologies Ltd., Hertfordshire, UK) in a 25 μL reaction volume containing a 10× PCR buffer (Invitrogen), 0.2 mM dNTPs (Invitrogen), 0.4 mM of each primer, 2 mM MgCl_2_ (Invitrogen), 20 ng of extracted DNA and 1 U Taq DNA polymerase (Invitrogen). The PCR conditions used by Bhavana et al. [18] were as follows: initial denaturation at 94 °C for 2 min, followed by 35 cycles of denaturation at 94 °C for 30 s, annealing at 56 °C for 30 s and elongation at 72 °C for 1 min, then a final extension at 72 °C for 5 min. To check for the presence of amplified products, 3 μL of the PCR products mixed with an equal amount of a 6× loading dye with GelRed (Biotium) was dispensed into wells of 1.5% agarose gel in a 1× Tris–borate–EDTA buffer and was subjected to electrophoresis at 100 V for 30 min. A 1 kbp plus ladder saturated with loading dye was used as the marker DNA. Next, the agarose gel was viewed using the Axygen Gel Documentation System (Corning Inc., Amsterdam, The Netherlands) to check for the presence of bands. The PCR products for each sample were sent to Apical Scientific Sdn. Bhd. (Malaysia) for DNA sequencing. A consensus from full sequences derived from the forward and reverse DNA sequences of each sample was obtained using the ClustalW Multiple Alignment tool [27] of the sequence editing software BioEdit [28]. The identity of the consensus sequence was determined using the BLASTN program [29]. Phylogenetic analysis was performed using MEGA X [30] to determine the clustering of the consensus sequence with other Mi23 locus marker genomic sequences published in the NCBI’s Genbank. A phylogenetic tree was constructed using the neighbor-joining method and subjected to a bootstrapping test with 1000 replicates.

### 2.7. Biochemical Analysis

Tomato varieties were checked for their biochemical response against *M. incognita* infection. Roots of inoculated and uninoculated tomato plants were analyzed for their total antioxidant activity, total phenolic content and ascorbic acid content at 45 dpi. Root extracts were prepared by grinding 500 mg of root samples that had been oven-dried for 48 h, which were then transferred to test tubes containing 5 mL of 50% methanol, followed by mixing for 3–4 min using a vortex mixer. Another 5 mL of methanol was added, followed by filtration. Samples were kept in vials and stored in a refrigerator. For the analysis of total antioxidant activity (AOA), 1 mM of a fresh 2, 2-diphenyl-1-picrylhydrazyl (DPPH) solution was prepared by dissolving 4 mg of DPPH into 25 mL of methanol. The final volume was adjusted to 100 mL. Root extracts (0.5 mL) were transferred to small test tubes, and 2.5 mL of 1 mM DPPH was added. A blank sample was prepared by adding 3 mL of 1 mM DPPH to a small test tube. Tubes were incubated for 30 min under dark conditions, and the absorbance was read at 517 nm using a UV-VIS spectrophotometer (Shimadzu). To determine the percentage of relative scavenging activity (% RSA), the following formula was used:

$$\%\;\mathrm{RSA}=\left(\frac{\mathrm{Test}\;\mathrm{sample}\;\mathrm{absorbance}}{\mathrm{Blank}\;\mathrm{sample}\;\mathrm{absorbance}}\right)\times 100$$

The total phenolic (TP) contents of the samples were determined using a modified method from the study of Velioglu et al. [31]. Root extracts were mixed thoroughly with 0.5 mL of distilled water, 1.0 mL of 0.2 sodium carbonate and 0.2 mL of Folin–Ciocalteu phenol reagent with a vortex mixer and were incubated for 15 min in a boiling water bath. Tubes were cooled to room temperature, and the absorbance was read at 710 nm using a UV-VIS spectrophotometer (Shimadzu). A standard curve was prepared using gallic acid. Total phenolic content was calculated from the standard curve using an interpolation method.

The amount of ascorbic acid was determined using a modified method used by Jagota and Dani [32]. Oven-dried ground root samples (100 mg) were transferred to test tubes and extracted twice using 5 mL 10% trichloroacetic acid (TCA). The mixture was filtered, and the extracts were placed in an ice bath for 5 min. TCA extracts (1.0 mL) were transferred to small test tubes, and 0.5 mL of 10% Folin–Ciocalteu reagent was added. The samples were mixed and allowed to stand for 10 min. Absorbance was read at 760 nm using a UV-VIS spectrophotometer (Shimadzu). A standard curve was prepared using ascorbic acid, and the ascorbic acid content was calculated from the standard curve using an interpolation method. 

## 3. Results

### 3.1. Growth and Disease Reaction of Tomato Varieties Infected with Meloidogyne incognita

**Figure 1:**
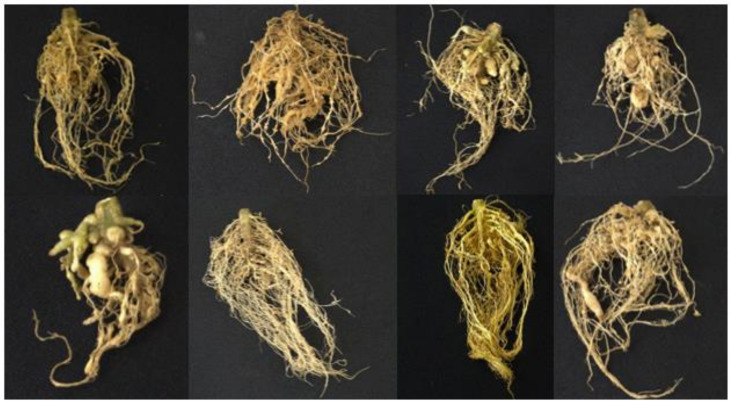
Disease reactions in the roots of tomato varieties infected with *Meloidogyne incognita*. Top (from left to right): Diamante Max F1, Ilocos Red, Avatar TY F1 and Athena; Bottom (from left to right): Rica, Tm 2016 11-1 Cherry, 2018-54 Cherry Tm LTB and Rosanna (the susceptible control).

The bioassay for resistance screening was conducted under net house conditions. The plant growth parameters (expressed in percentage of reduction), the number of galls and the gall indices of tomato varieties were assessed at 45 dpi (Table 1). Tomato varieties showed no significant differences in terms of the percentage of reduction in plant height. In contrast, an increase in fresh weight and shoot weight was noted in Tm 2016 11-1 Cherry, which was significant compared with Avatar TY F1 and Rica but not with the other varieties. There was an increase in root weight among the tomato varieties but they were not significantly different. The number of galls observed ranged from 49 (Athena) to 265 (Ilocos Red). Athena and Rica had a significantly lower number of galls when compared with Ilocos Red and 2018-54 Cherry LTB but not with the rest of the tomato varieties tested. All of the varieties were found to be susceptible to highly susceptible to *M. incognita* according to [31]. Heavy galling was observed, with some variation in the distribution and sizes among the tomato varieties when compared with Rosanna (the susceptible control) (Figure 1).

### 3.2. Mi23 Marker and Phylogenetic Analyses

**Figure 2:**
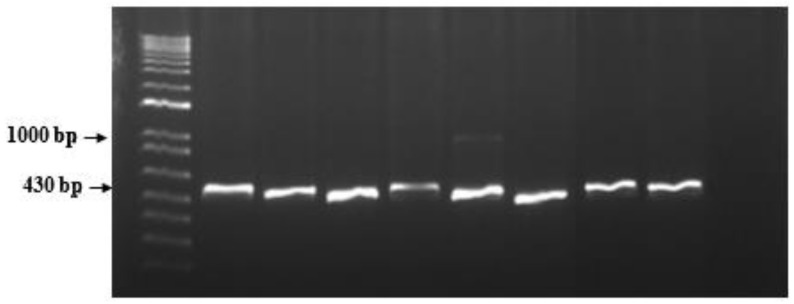
PCR product obtained using the Mi23 marker in different tomato varieties infected with *Meloidogyne incognita*. From left to right: 1 kb plus DNA ladder, Diamante Max F1, Ilocos Red, Rosanna (susceptible control), Athena, Avatar TY F1, Rica, Tm 2016 11-1 Cherry and 2018-54 Cherry Tm LTB. The amplified band has a fragment size of 430 bp.

Through PCR, the molecular marker Mi23 was used to detect the presence of the *Mi-1* gene in the tomato varieties. PCR with Mi23 yielded 430 bp fragments in all tomato varieties tested. In addition, an extra band of approximately 1000 bp was observed in Avatar TY F1 (Figure 2). The PCR-amplified products (430 bp) from the samples were sequenced. Through use of the BLASTN program from the NCBI, the consensus sequence derived from the sequences of the 430 bp PCR product from the tomato varieties showed that it was within chromosome 6 of *S. lycopersicum* and had 100% similarity to the Mi23 locus marker genomic sequence of *S. lycopersicum* cultivar M82-1-8 (accession number: EU033927.1).

**Figure 3:**
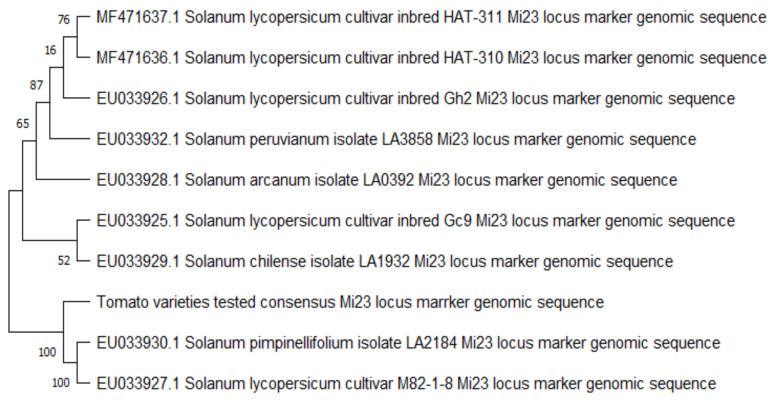
Phylogenetic analysis using the neighbor-joining method, showing the clustering of the consensus genomic sequence obtained using the Mi23 locus marker in the tested tomato varieties, along with reference sequences of tomato genotypes and wild relatives reported to have resistance or susceptibility to root-knot nematodes.

Phylogenetic analysis through the neighbor-joining method using the consensus sequence from the tomato varieties and reference sequences obtained using the Mi23 marker in tomato and its wild relatives with or without resistance to root-knot nematodes showed two major clusters (Figure 3). Cluster A included the tomato genotypes and the wild relatives of tomato with reported resistance against the root-knot nematode *M. incognita*, while Cluster B has the consensus sequence of the tomato varieties, grouped with a subcluster formed by the wild relative *S. pimpinellifolium* isolate LA2184 (accession number: EU33930.1) and the *S. lycopersicum* cultivar M82 1-8. In Cluster A, the *S. lycopersicum* cultivar inbred Gc9 and the *S. chilense* isolate LA1932 formed a separate subcluster. The rest of the tomato genotypes and the wild relatives were grouped together in another subcluster which included the *S. lycopersicum* cultivar inbreds HAT-310 (accession number: MF471636.1), HAT-311 (accession number: MF471637.1) and Gh2 (accession number: EU33926.1), and the *S. peruvianum* isolate LA3858 (accession number: EU033932.1) and the *S. arcanum* isolate LA0392 (accession number: EU033928.1). 

### 3.3. Biochemical Response of Tomato Varieties to Infection by Meloidogyne incognita 

**Figure 4:**
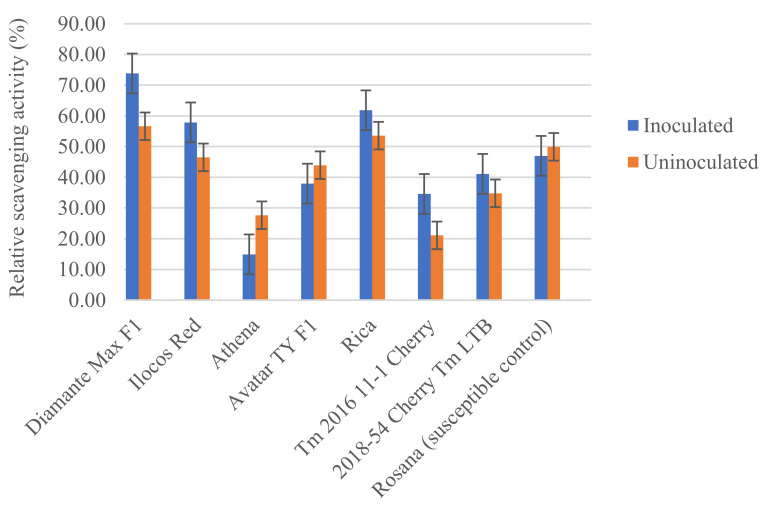
Average relative scavenging activity of extracts from different tomato varieties tested against *Meloidogyne incognita*. Error bars represent standard error of the mean.

Biochemical analysis revealed varying responses among the tomato varieties when tested against *M. incognita* at 45 dpi. The chemical properties of roots related to the resistance reaction, namely total antioxidant activity, total phenolic content and ascorbic acid content, were determined in both inoculated and uninoculated tomato plants. Analysis of the total antioxidant activity using the DPPH method revealed the lowest activity in extracts from Athena (14.91% and 27.65%), while the highest activity was observed in Diamante Max F1 (73.83% and 56.62%). No differences were observed among Ilocos Red, Rica and Rosanna, while the same level of antioxidant activity was found in Tm 2016 11-1 Cherry and 2018-54 Cherry Tm LTB. Total AOA in Diamante Max F1, Ilocos Red and Tm 2016 11-1 Cherry increased by an average of 16.21% when inoculated with root-knot nematodes compared with the uninoculated control. In contrast, the tomato variety Athena had a reduction in antioxidant activity of 12.74% when inoculated with *M. incognita*. No differences were observed between inoculated and uninoculated plants in other tomato varieties (Figure 4). 

**Figure 5:**
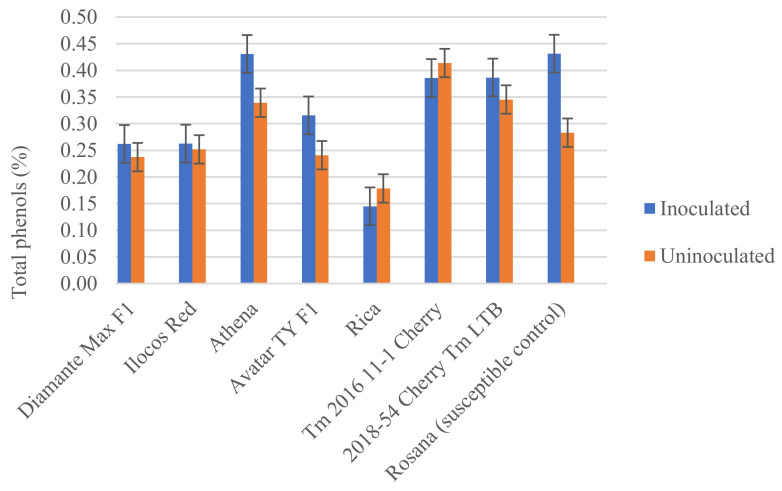
Average total phenols in gallic acid equivalents of extracts from different tomato varieties tested against *Meloidogyne incognita.* Error bars represent standard error of the mean.

Total phenols extracted from the tomato varieties tested with *M. incognita* showed varying levels (Figure 5). The lowest amount of TP was recorded in Rica (0.14 and 0.18% in GAE), while the highest amount was observed in Athena (0.43 and 0.34% in GAE) and Rosanna (0.43 and 0.28% in GAE) in the inoculated and uninoculated plants. Tm 2016 11-1 Cherry and 2018-54 Cherry Tm LTB showed similar levels of TP after Athena and Rosanna. These were followed by Avatar TY F1, Diamante Max F1 and Ilocos Red. An increase in TP by an average of 0.11% in GAE was observed in Athena, Avatar TY F1 and Rosanna when inoculated with root-knot nematodes compared with the uninoculated counterparts. In contrast, no differences were recorded in Diamante Max F1, Ilocos Red, Rica, Tm 2016 11-1 Cherry and 2018-54 Cherry Tm LTB between inoculated and uninoculated plants.

**Figure 6:**
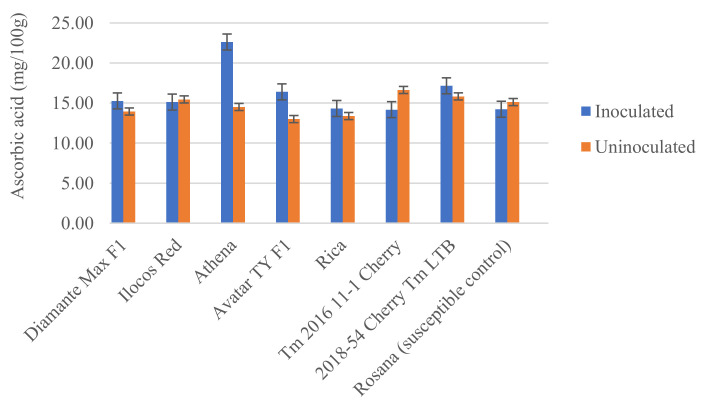
Average ascorbic acid of extracts from different tomato varieties tested against *Meloidogyne incognita*. Error bars represent standard error of the mean.

The response of the tomato varieties based on their production of ascorbic acid was also determined. Ascorbic acid levels increased by an average of 5.76 mg/100 g when inoculated with *M. incognita* in Athena and Avatar TY F1 compared with their corresponding uninoculated counterparts. No difference was observed between the inoculated and uninoculated plants for the other varieties, except for Tm 2016 11-1 Cherry, where a reduction of 2.4 mg/100 g was observed when inoculated with *M. incognita* (Figure 6). 

## 4. Discussion

This study showed that there is no resistance in the selected germplasm collections and local tomato cultivars against the root-knot nematode *M. incognita*. Instead, susceptible to hypersusceptible reactions were observed in the tomato varieties. There was no significant difference in the growth parameters among the varieties tested, except for Tm 2016 11-1 compared with Rica. Generally, there was an increase in root weight in all the varieties screened. This can be attributed to gall formation in the tomato varieties, which varied in shape and size. The number of galls was significantly lower in Athena and Rica compared with Ilocos Red and 2018-54 Cherry LTB, but not with rest of the varieties. 

This was confirmed by using the SCAR marker Mi23 to detect the presence of the *Mi-1* gene conferring resistance against RKN. PCR yielded a 430 bp fragment in all of the varieties screened, which corresponded to the reported fragment obtained from susceptible genotypes [17,18,19]. Moreover, an extra fragment approximately 1000 bp in size was observed in Avatar TY F1. BLAST analysis revealed that the 430 bp fragment obtained from the varieties tested had 100% similarity with the *S. lycopersicum* cultivar M82-1-8, which was used as the susceptible check by Garcia et al. [33] in an evaluation of the Mi23 marker for detecting the *Mi-1* locus. Phylogenetic analysis using the neighbor-joining method showed that the consensus sequence from the tomato varieties grouped with the *S. pimpinellifolium* isolate LA2184 and *S. lycopersicum* cv. M82-1-8. In contrast, a different cluster was formed by reference sequences of the *S. peruvianum* isolate LA3858, the *S. arcanum* isolate LA0392, and the *S. lycopersicum* cultivar inbreds Gh2, HAT-310 and HAT 311, which have reported resistance against root-knot nematodes [18,19]. Interestingly, this cluster also included the reference sequences of the *S. lycopersium* cultivar inbred Gc9 and the *S. chilense* isolate LA1932, but these were grouped in a separate subcluster. Both have resistance against begomoviruses but are susceptible to root-knot nematodes [19].

Biochemical analysis showed the varying reactions of the tomato varieties to RKN infection. This study showed differences in the basal level of total antioxidant activity among the tomato varieties. While an increase in total antioxidant activity was observed in the resistant genotype, as reported by Chawla et al. [14], an increase in the total antioxidant activity by an average of 16.21% in Diamante Max F1, Ilocos Red and Tm 2016 11-1 Cherry and a reduction of 12.74% in Athena were observed in this study. Chawla et al. [14] reported an increase in total AOA in RKN-resistant genotypes. In contrast, this study showed that an increase in total AOA after RKN infection may not translate to a resistance reaction. Hence, the timing and localization seemed to be important. Total phenols in each of the tested varieties were also determined. the basal level of TP also varied among the tomato varieties. Athena, Avatar TY F1 and Rosanna showed an average increase in TP of 0.11% in GAE when inoculated with *M. incognita*. This study concurred with the study by Giebel [16], where an increase in total phenols was also observed in susceptible genotypes when infected with *M. incognita* and not only in resistant genotypes. Bajaj and Mahajan [34] reported that similar compositions of phenols can be produced by resistant and susceptible genotypes but not similar concentrations. Lastly, ascorbic acid levels were also determined in the tomato varieties. An average increase of 5.76 mg/100 g in Athena and Avatar TY F1 and a reduction of 2.4 mg/100 g in Tm 2016 11-1 Cherry were observed when inoculated with *M. incognita*. Arrigoni et al. [12] reported that the resistance reaction against *M. incognita* is accompanied by an increase in the ascorbic acid concentration while susceptible plants remained unaltered. Further, a gradual increase could be observed in the first 12 days of infection, which then declined back to its initial level. This study showed that increase in ascorbic acid did not translate to a resistance reaction, as observed in Athena and Avatar TY F1. In addition, levels of ascorbic acid can remain elevated even at 45 dpi. 

## 5. Conclusions

This study revealed that the selected tomato germplasm collections and commercially available cultivars have no resistance against the root-knot nematode *M. incognita* according to their growth responses and disease reactions, their lack of the *Mi-*1 gene conferring resistance against RKN and their biochemical response to RKN infection. Thus, this study showed the vulnerability of local tomato growers to threats posed by RKN infection. In addition, it also shows the need to identify possible sources of resistance against *M. incognita* and produce resistant tomato varieties. 

## Figures and Tables

**Table 1:** Effects on plant growth, the number of root galls and the gall index of tomato varieties infected with *Meloidogyne incognita*.

Variety	Plant Height	Fresh Weight	Shoot Weight	Root Weight	Number of Galls	Gall Index
Diamante Max F1	−31.2 ^a^	−22.9 ^ab^	−30.5 ^ab^	76.7 ^a^	189.8 ^abc^	4.8 ^ab^
Ilocos Red	−22.8 ^a^	−3.9 ^ab^	−13.4 ^ab^	145.6 ^a^	265.3 ^a^	5.0 ^a^
Avatar TY F1	−32.5 ^a^	−53.2 ^a^	−58.8 ^a^	92.4 ^a^	133.3 ^bcd^	4.8 ^ab^
Athena	−23.6 ^a^	−32.2 ^ab^	−40.2 ^ab^	122.2 ^a^	48.5 ^d^	3.8 ^b^
Rica	−44.9 ^a^	−60.6 ^a^	−67.9 ^a^	66.7 ^a^	54.8 ^d^	4.0 ^b^
Tm 2016 11−1 Cherry	−3.2 ^a^	30.0 ^b^	22.0 ^b^	216.7 ^a^	94.3 ^cd^	4.5 ^ab^
2018−54 Cherry Tm LTB	−18.7 ^a^	−18.0 ^ab^	−28.6 ^ab^	135.6 ^a^	255.8 ^ab^	5.0 ^a^
Rosanna (susceptible control)	−9.3 ^a^	−3.4 ^ab^	−10.2 ^ab^	117.7 ^a^	122.7 ^cd^	4.5 ^ab^

*Meloidogyne incognita* juveniles (J2s) were inoculated into several tomato varieties: Diamante Max F1, Ilocos Red, Avatar TY F1, Athena, Rica, Tm 2016 11-1 Cherry, 2018-54 Cherry Tm LTB and Rosanna (the susceptible control). The growth parameters and galling were assessed at 45 dpi. Values are means (N = 6) of the percentage of reduction in the growth parameters, as indicated by a (−) sign. Different letters in different rows indicate significant differences from a one-way ANOVA with post-hoc Tukey’s HSD test, while the Kruskal–Wallis test with Dunn’s test was used for the galling indices (both *p* ≤ 0.05). Gall indices were determined using a visual rating scale with a range of 0–5 according to Taylor and Sasser (1978) (0 = no gall or no infection (immune); 1 = 1–2 galls (highly resistant); 2 = 3–10 galls (resistant), 3 = 11–30 galls (moderately resistant); 4 = 31–100 galls (susceptible); and 5 = 100 or more galls (highly susceptible).

## Data Availability

The data that support the findings of this study are openly available.

## References

[1] Food and Agriculture Organization Corporate Statistical Database (2017). FAOSTAT: Crops. Food Agriculture Organization, United Nations. https://www.fao.org/faostat/en/#home.

[2] Philippines Statistics Authority (2017). Costs and Returns of Tomato Production.

[3] Sikora R.A., Fernandez E., Luc M., Sikora R.A., Bridge J. (2005). Nematode Parasites of Vegetables. Plant Parasitic Nematodes in Tropical and Subtropical Agriculture.

[4] Mitkowski N.A., Abawi G.S. (2003). Root-knot nematodes. Plant Health Instr..

[5] Valdez R.B. (1978). Nematodes Attacking Tomato and Their Control. Proceedings of the 1st International Symposium on Tropical Tomato.

[6] Madamba C.P. (1981). Distribution and identification of Meloidogyne spp. in the Philippines and five other Asian countries. Philipp. Agric..

[7] Davide R.G. (1988). Nematode problems affecting agriculture in the Philippines. J. Nematol..

[8] Roberts P.A., May D., Matthews W.C. (1986). Root-knot nematode resistance in processing tomatoes. Calif. Agric..

[9] Milligan S.B., Bodeau J., Yaghoobi J., Kaloshian I., Zabel P., Williamson V.M. (1998). The Root knot nematode resistance gene Mi from tomato is a member of the leucine zipper, nucleotide binding, leucine-rich repeat family of plant genes. Plant Cell.

[10] Smith P.G. (1944). Embryo culture of a tomato species hybrid. Proc. Am. Soc. Hortic. Sci..

[11] Medina Filho H.P., Stevens M.A. (1980). Tomato breeding for nematode resistance: Survey of resistant varieties for horticultural characteristics and genotype of acid phosphates. Acta Hortic..

[12] Arrigoni O., Zacheo G., Arrigoni-Liso R., Bleve-Zacheo T., Lamberti F. (1979). Relationship between ascorbic acid and resistance in tomato plants to *Meloidogyne incognita*. Phytopathology.

[13] Branch C., Hwang C.-F., Navarre D.A., Williamson V.M. (2007). Salicylic acid is part of the Mi-1-mediated defense response to root-knot nematode in tomato. Mol. Plant Microbe Interact..

[14] Chawla N., Choudhary K., Kaur S., Jindal S. (2013). Changes in antioxidative enzymes in resistant and susceptible genotypes of tomato infected with root-knot nematode (*Meloidogyne incognita*). Indian J. Nematol..

[15] Cooper W.R., Jia L., Goggin L. (2005). Effect of jasmonate-induced defenses on root-knot nematode infection of resistant and susceptible tomato cultivars. J. Chem. Ecol..

[16] Giebel J. (1966). Phenolic content in roots of some Solanaceae and its influence on IAA-oxidase activity as an indication of resistance to *Heterodera rostochiensis*. Nematologica.

[17] Devran Z., Başköylü B., Taner A., Doǧan F. (2013). Comparison of PCR-based molecular markers for identification of *Mi* gene. Acta Agric. Scand. Sect. B Soil Plant Sci..

[18] Bhavana P., Singh A.K., Kumar R., Prajapati G.K., Thamilarasi K., Manickam R., Maurya S. (2018). Identification of resistance in tomato against root knot nematode (*Meloidogyne incognita*) and comparison of molecular markers for *Mi* gene. Australas. Plant Pathol..

[19] Seah S., Williamson V.M., Garcia B.E., Mejia L., Salus M.S., Martin C.T., Maxwell D.P. (2007). Evaluation of a co-dominant SCAR marker for detection of the Mi-1 locus for resistance to root-knot nematode in tomato germplasm. Tomato Genet. Coop. Rep..

[20] Davide R.G., Sasser J.N., Carter C.C. (1985). Summary Report on the Current Status, Progress and Needs for *Meloidogyne* Research in Region VI. An Advance Treatise on Meloidogyne.

[21] Eisenback J.D., Sasser J.N., Carter C.C. (1985). Diagnostic Characters Useful in the Identification of the Four Most Common Species of Root-Knot Nematodes (*Meloidogyne spp*.). An Advance Treatise on Meloidogyne.

[22] Hartman K.M., Sasser J.N., Barker K.R., Carter C.C., Sasser J.N. (1985). Identification of Meloidogyne species on the Basisi of Differential Host Test and Perineal-Pattern Morphology. An Advance Treatise on Meloidogyne.

[23] Coyne D.L., Ross J.L. (2014). Nematode Resistance Screening Root-Knot Nematodes Meloidogyne spp..

[24] Taylor A.L., Sasser J.N. (1978). Biology, Identification and Control of Root-Knot Nematodes (Meloidogyne spp.).

[25] Fox J., Bouchet-Valat M. Rcmdr: R Commander. R Packag.

[26] Doyle J.J., Doyle J.L. (1990). Isolation of plant DNA from fresh tissue. Focus.

[27] Thompson J.D., Higgins D.G., Gibson T.J. (1994). CLUSTAL W: Improving the sensitivity of progressive multiple sequence alignment through sequence weighting, position-specific gap penalties and weight matrix choice. Nucleic Acid Res..

[28] Hall T.A. (1999). BioEdit: A user-friendly biological sequence alignment editor and analysis program for Windows 95/98/NT. Nucleic Acids Symp. Ser..

[29] Zhang Z., Schwartz S., Wagner L., Miller W. (2000). A greedy algorithm for aligning DNA sequences. J. Comput. Biol..

[30] Kumar S., Stecher G., Li M., Knyaz C., Tamura K. (2018). MEGA X: Molecular Evolutionary Genetics Analysis across computing platforms. Mol. Biol. Evol..

[31] Velioglu Y.S., Mazza G., Gao L., Oomah B.D. (1998). Antioxidant Activity and Total Phenolics in Selected Fruits, Vegetables, and Grain Products. J. Agric. Food Chem..

[32] Jagota S.K., Dani H.M. (1982). A new calorimetric technique for the estimation of vitamin C using folin phenol reagent. Anal. Biochem..

[33] Garcia B.E., Mejia L., Salus M.S., Martin C.T., Seah S., Williamson V.M., Maxwell D.P. (2007). A Co-Dominant SCAR Marker, Mi23, for Detection of the Mi-1.2 Gene for Resistance to Root-Knot Nematode in Tomato Germplasm. http://invirlab.plantpath.wisc.edu/GeminivirusResistantTomatoes/Markers/MAS-Protocols/Mi23-SCAR.pdf.

[34] Bajaj K.L., Mahajan R. (1977). Phenolic compounds in tomato susceptible and resistant to *Meloidogyne incognita* (Kofoid et White) Chitwood. Nematol. Mediterr..

